# An observational retrospective study of odontogenic cyst´s and tumours over an 18-year period in a Portuguese population according to the new WHO Head and Neck Tumour classification

**DOI:** 10.4317/medoral.24337

**Published:** 2020-12-19

**Authors:** Luis Monteiro, Catarina Santiago, Barbas do Amaral, Azza Al-Mossallami, Rui Albuquerque, Carlos Lopes

**Affiliations:** 1 Oral Medicine and Oral Surgery Department, University Institute of Health Sciences (IUCS), Gandra, Portugal; 2 nstituto de Investigação e Formação Avançada em Ciências e Tecnologias da Saúde (IINFACTS), University Institute of Health Sciencies (IUCS), Gandra, Portugal; 3 Stomatology Department, Hospital de Santo António - Porto Hospitalar Center (CHP), Portugal; 4 Oral Surgery Department, Guy's and St Thomas Hospital NHS Foundation Trust, London, United Kingdom; 5 Oral Medicine Department, Guy's and St Thomas Hospital NHS Foundation Trust; King's college London, United Kingdom; 6 Pathology and Immunology Department, Porto Hospitalar Center (CHP) and Institute of Biomedical Sciences Abel Salazar (ICBAS), Porto University, Portugal

## Abstract

**Background:**

Odontogenic cysts and tumours of the jaws represent one of the most prevalent groups of oral-maxillofacial lesions. We aimed to evaluate the clinical and pathological characteristics of a cohort of odontogenic cysts (OC) and odontogenic tumours (OT) of the jaws in a Portuguese population.

**Material and Methods:**

This observational retrospective study analysed patients diagnosed with either an OC or OT of the jaws at a central hospital of Oporto, Portugal, between 1988 and 2006. Data collected from patients’ files included demographic, clinical, radiological and histopathological information. Recurrence was evaluated using univariate and multivariate analysis.

**Results:**

The sample consisted of 397 patients, 231 males (58.2%) and 166 females (41.8%), with a mean-age of 36.7±17 years. Twenty-seven patients (6.8%) presented with more than one lesion providing a total of 433 lesions. There were 396 (91.5%) OC, mostly represented by radicular cysts (n=257;59.4%), dentigerous cysts (n=79;18.2%), or odontogenic keratocysts (n=50;11.5%). There were 37 (8.5%) OT, mostly represented by ameloblastomas (n=16;3.7%), and odontomas (n=9;2.1%). The most common initial clinical manifestation was swelling (n=224;51.7%). Recurrence was observed in 30 cases (6.9%), mostly in ameloblastomas (n=6;37.5%) and odontogenic keratocysts (n=12;24%). In the multivariate analysis the diagnosis classification of the lesion was the only independent and significant variable related with the recurrence (*P*=0.04).

**Conclusions:**

Radicular cysts were the most commonly occurring type of OC and ameloblastomas the most commonly occurring OT. Amelobastomas and odontogenic keratocysts were the lesions with the highest rates of recurrence. This large sample provides useful information about the frequency profile and characteristics of OC and OT over a period of 18 years, allowing valuable comparison with data from other countries.

** Key words:**Odontogenic cysts and tumours, radicular cyst, dentigerous cyst, odontogenic keratocyst, ameloblastoma, recurrence.

## Introduction

Odontogenic cysts and tumours of the jaws represent one of the most prevalent groups of oral-maxillofacial lesions (1). Jaw cysts, firstly reported by Fauchard in 1728, can be defined as intraosseous pathological cavities filled with fluid, semi-solid or gaseous material, partially or fully covered over with epithelial tissue, and bounded by a capsule of connective tissue. When the cyst has developed from odontogenic epithelial remnant tissues, it is designated as an odontogenic cyst (OC). This category is then subdivided according to their inflammatory or developmental origin (2-4). Odontogenic tumours (OT) are first described in the scientific literature in 1869, when Pierre Paul Broca introduced the term "odontoma" to classify a tumorous lesion originating from tooth-forming tissues (4). They correspond to a heterogeneous group of lesions ranging from hamartomas, to neoplastic or malignant lesions derived from ectodermal and/ or mesenchymal odontogenic tissues (5).

Taking into account the differing nature of odontogenic cysts and tumours, several consensus meetings have tried to produce a more systematic classification of these lesions as promoted by the World Health Organization (WHO) in 1971(4). Since then, other WHO meetings have revised the classifications of these lesions in 1992, 2005 and 2017 (6). One of the biggest changes in classification was reported in the 3rd edition of the Odontogenic Tumour Classification in 2005, where odontogenic keratocysts were considered neoplasm tumours, and referred to as keratocystic odontogenic tumours (KOT). This was to take into account the behaviour of the lesion, histopathology, genetic factors and its tendency for being an aggressive and recurrent lesion (6). However, the controversy over odontogenic tumour classifications continued. Reports of successful conservative treatment of keratocystic odontogenic tumours (with decompression only) questioned the true neoplastic nature of these lesions (6). Furthermore, several reports have documented an increase in the prevalence of odontogenic tumours as a result of this classification change (7,8). In 2017, the new WHO classification of odontogenic tumours reclassified KOT as odontogenic keratocysts, a developmental cyst (6).

Although several odontogenic cysts and tumours case series have been reported worldwide, few of them have evaluated and compared both groups of lesions together (7-9), as well as having information on recurrence (2,10-14), and using longitudinal analysis.

Our aims were to analyse the frequency profile of odontogenic cysts and tumours of the jaws in a Portuguese population using the new WHO classification system and to evaluate their clinical-pathological characteristics with a focus on a longitudinal analysis of recurrence.

## Material and Methods

An observational retrospective study was carried out on a cohort of patients who had been diagnosed with odontogenic cysts or odontogenic tumours between 1988 and 2006 from the Pathology Department of “Centro Hospitalar do Porto” (CHP), Portugal. The study was reviewed and approved by the institutional review board of the hospital (Investigation, Formation and Teaching Department – DEFI; 024/CES/03). The study was performed in full accordance with the World Medical Association Declaration of Helsinki.

The patients’ clinical files and imaging exams were reviewed and analysed for data collection. Data on several variables was collected, as follows: age, gender, social history and habits (e.g. smoking and alcohol consumption), initial clinical manifestations, evolution of the lesion (in months), anatomic location (i.e. maxilla or mandible; anterior or posterior; anterior was defined as being from the incisor to canine region and posterior was defined as extending from the premolars posteriorly to include the molars, mandibular ramus or maxillary sinus and tuberosity), radiographic characteristics such as appearance and shape (classified as radiolucent, or radiopaque, and unilocular or multilocular), size (largest diameter on panoramic radiograph in centimetres), and association to adjacent teeth (inclusion or impacted, tooth displacement or root resorption), clinical and histopathological diagnosis, treatment performed, follow-up information and the presence of recurrence.

For the review of histological characteristics and diagnosis, we performed a review of the histopathological slides (4-µm) that were stained with hematoxylin/eosin (HE). The histological classification was re-evaluated according to the 2017 World Health Organization (WHO) classification of Head and Neck Tumours by two independent observers.

After reviewing all available information, we included cases with a clinical/radiographic and histopathological diagnosis of primary OC or OT in the maxillo-facial region, irrespective of age or gender. We excluded non-primary cases, cases without a histopathological diagnosis or with insufficient clinical data for the diagnosis. The initial sample comprised a total of 484 patients, 87 cases were excluded due to the absence of appropriate imaging or inconsistent/ insufficient data to characterise the lesions, leading to a final sample of 397 patients with a total of 433 lesions.

- Statistical analysis

The statistical analysis was performed using the IBM SPSS software (Statistical Package for the Social Sciences) version 24.0® (IBM Corporation, NY, US). The results are presented in absolute and relative frequencies. Chi-square test and Fisher's exact test (in case of non-valid chi-square test) were used to evaluate the associations between categorical variables. Continuum variables were analysed using Anova test. Recurrence-free time interval was defined as the time interval (in months) between diagnosis and the first histological confirmed recurrence and evaluated using Kaplan-Meier curves with log-rank test. Variables that were significant at univariate analysis were included into a multivariate analysis using the Cox proportional hazards model method. The significance level used was *P*<0.05.

## Results

- General Description of Sample

Of the 397 included patients, 231 were males (58.2%) and 166 females (41.8%), ranging from 5 to 89 years-old, with a mean age of 36.7±17 years. A high proportion of cases presented in the third (n=105; 26.4%) and fourth (n=92; 23.2%) decades of life. Most of the patients presented with a single lesion (n=370; 93.2%) at diagnosis, and 27 (6.8%) presented with more than one lesion (OC or OT) providing a final sample of 433 lesions in total. Other variables are listed in Table 1.

Taking into account clinical, imaging and histological aspects, 396 cases were classified as odontogenic cysts (91.5%) and 37 cases (8.5%) as odontogenic tumours. There was no statistical difference between gender (*P*=0.959) and age (*P*=0.133) regarding OC and OT groups.

The most affected jaw was the maxilla (n=240; 55.4%), in particular the anterior region (n=93; 38.8%). For mandibular cases (n=193;44.6%), the posterior region was the area most predominantly affected (n= 138;71.5%) (*P*<0.001) (Table 1).

Most of the lesions were radiolucent (n=421, 97.5%), and unilocular (n=404; 96%). All OC cases presented a radiolucent radiographic image (n=396; 100%). The mean radiographic size was 3.3±1.7cm (range 1-12cm). 113 cases (26.1%) involved included teeth adjacent to the lesions; 99 cases of OC (25%) and 14 of OT (37.8%) (*P*=0.089). Other radiograph characteristics distributed by the type of lesion are outlined in Table 1.

The most common initial clinical manifestation that lead patients to seek medical help was swelling/enlargement (n=224; 51.7%). This was proportionally higher amongst the OT cohort, occurring in 70.3% of cases (n= 26) compared to 50% of OC cases (n=198). Other clinical manifestations are listed in Table 1.

The treatment modality most commonly adopted was enucleation (n=425;98.2%), followed by partial resection (n=5; 1.2%) of the lesion, or by total resection (segmentary resection or hemi-mandibulectomy) (n=3; 0.7%). Overall recurrence was observed in 30 cases (6.9%), 23 of these cases were OC (5.8%) and 7 (18.9%) were OT cases (*P*=0.003).

**Figure 1:**
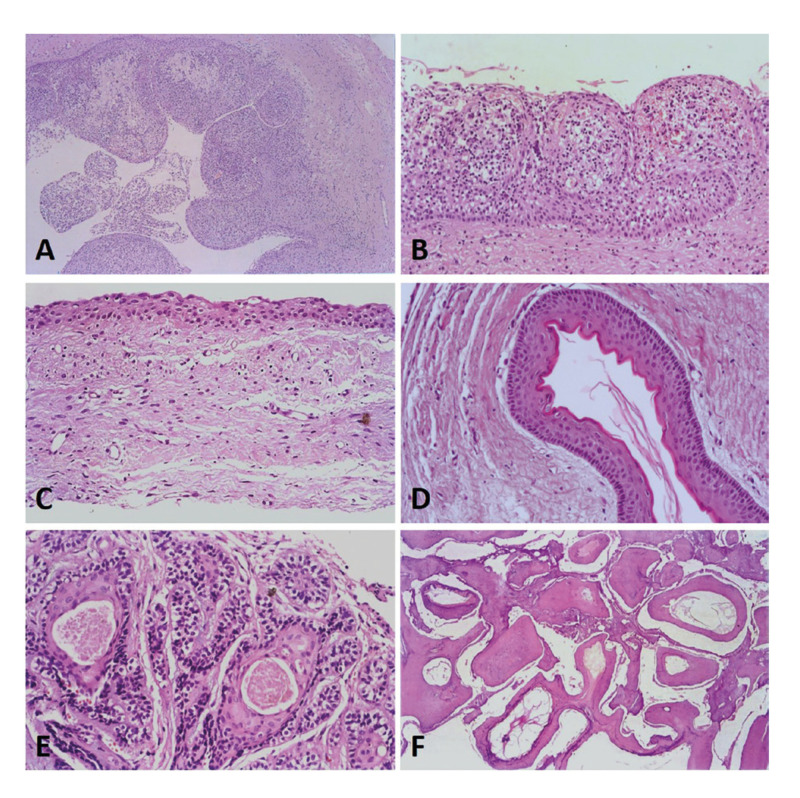
Histologic pictures (haematoxylin and eosin stain) from the 5 most common lesions found in the present sample: A - radicular cyst (at magnification of 5x); B – particular aspect of an epithelial lining of a radicular cyst (at magnification of 20x); C - dentigerous cyst (at magnification of 20x); D - odontogenic keratocyst (at magnification of 20x); E - ameloblastoma (acanthomatous subtype) (at magnification of 20x); and F - complex type odontoma (at magnification of 5x).

**Figure 2:**
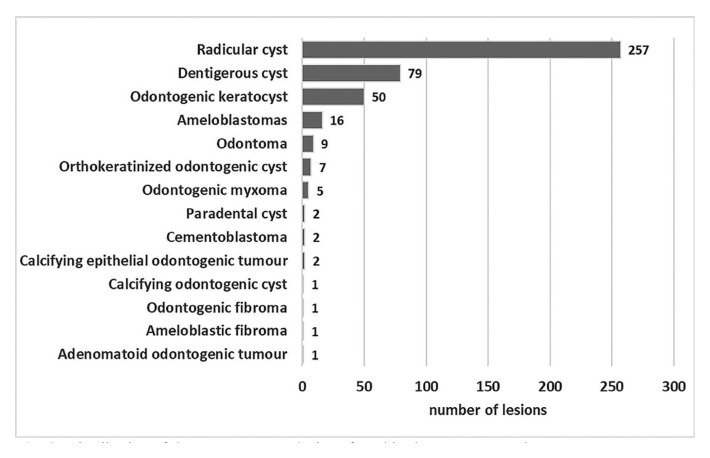
Distribution of the most common lesions found in the present sample.

When we analysed the diagnoses of the lesions, the most commonly occurring lesion was radicular cysts (n=257;59.4%), followed by dentigerous cysts (n=79;18.2%), odontogenic keratocysts (n=50; 11.5%), ameloblastomas (n=16; 3.7%), and odontomas (n=9; 2.1%) representing 95% of all lesions (Fig. 1, Fig. 2). In view of this we have carried out a comparative analysis of the 5 most common lesions in our cohort sample, which is discussed in the latter part of this paper.

**Table 1:**
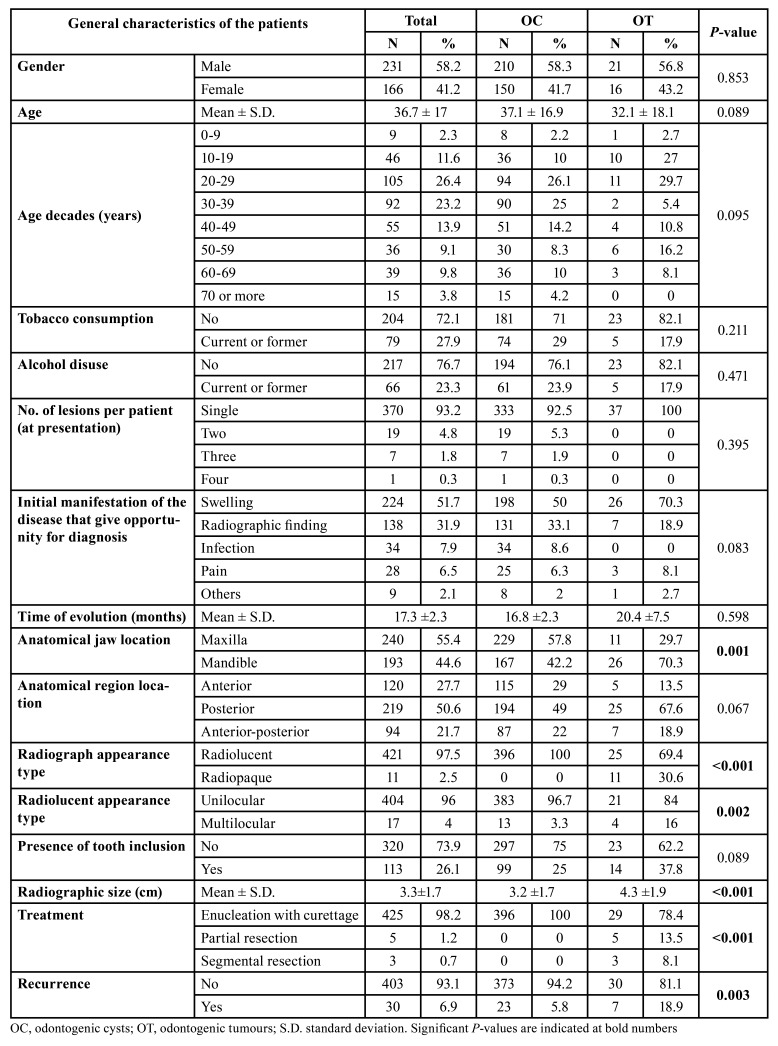
General characteristics of the patients with odontogenic cysts and odontogenic tumours.

Several subtypes were included in the main diagnosis. For instance, from the 16 ameloblastomas, 8 (50%) were solid type, 7 were (43.7%) unicystic and one (6.3%) was a peripherical type. With regards to the histological pattern of the ameloblastomas, there were 10 (62.5%) plexiform cases, one (6.3%) follicular, one (6.3%) acanthomatous and 4 mixed (25%) cases. From the 9 odontomas, 7 (77%) were complex and 2 (23%) were compound type. There were 35 (8.1%) residual cysts included in the diagnosis of radicular cysts, and 5 (1.2%) eruption cysts included in the dentigerous cyst diagnosis.

- Analysis of the 5 most common lesions

Age and Gender

**Figure 3:**
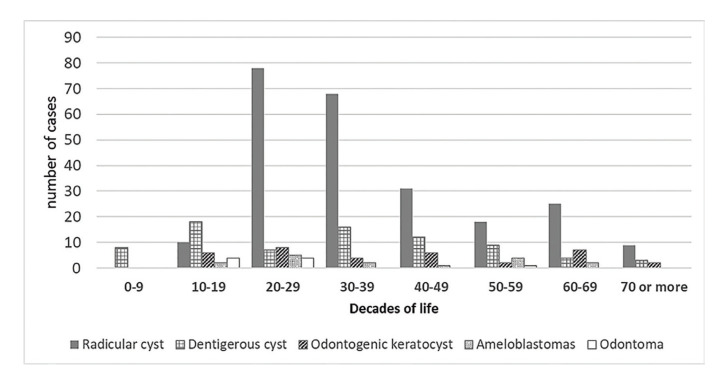
Distribution of the 5 most common lesions found in the present sample by age decades.

The mean age of patients with odontomas (22.9±12.5 years) and dentigerous cysts (33±19.5 years) were lower than those with radicular cysts (38.1±15.3 years), odontogenic keratocysts (39.3±19.8 years) and ameloblastomas (38.5±18.3 years) (*P*=0.015). Most of the lesions affected patients in their third and four decades of life (Fig. 3).

Dentigerous cysts were the only lesions that occurred in the patients’ first decade of life (n=8; 10.4%). Of these, 4 of them were eruption cysts (Table 2). The lesions that occurred most commonly in male patients were radicular cysts (n=137 ;57.3%), dentigerous cysts (n=53; 68.8%), and ameloblastomas (n=12; 75.0%). In comparison, odontogenic keratocysts (n=20; 57.1%) and odontomas (n=5; 55.6%) occurred more frequently in female patients (*P*=0.048) (Table 2).

Number of Lesions and Anatomical Site

The presence of multiple lesions occurred in cases of odontogenic keratocysts (10;28.6%), radicular cysts (13;5.4%) and dentigerous cysts (3;3.9%). All ameloblastomas and odontomas, in our sample, were single lesions (*P*<0.001). There were 6 cases of Gorlin-Goltz Syndrome amongst patients who had a diagnosis of odontogenic keratocysts (17.1% of the 35 patients diagnosed with odontogenic keratocysts). All syndromic patients presented with more than one odontogenic keratocyst at diagnosis and four (13.8%) non-syndromic patients also presented with multiple odontogenic keratocysts at diagnosis (*P*<0.001). Other manifestations of this syndrome included bone/skeletal abnormalities (spina bifida, pectus excavatum, scoliosis, frontal bossing, or hypertelorism), falx cerebri calcification, congenital cataracts, basal cell nevus and/or carcinomas as well as a family history of the disease (present in 3 of the 6 Gorlin-Goltz patients in our sample).

The mandible was the jaw most affected by dentigerous cysts (n=46; 58.2%), odontogenic keratocysts (n=42; 84%), ameloblastomas (n=14; 87.5%) and odontomas (n=5; 55.6%). Radicular cysts occurred mostly in the maxilla (n=187; 72.8%) (*P*<0.001). All compound odontomas were located in the maxilla and 5 (71.4%) of the 7 complex odontomas were locate in the mandible (*P*=0.073) (Table 3). The posterior region was predominantly affected in all 5 lesions (Table 2). Albeit this, maxillary radicular cysts were more common in the anterior region (80; 42.8%)(P<0.001).

Clinical Manifestation, Radiographic Findings and Diagnosis

Swelling was the first clinical manifestation that lead to detection of the lesions especially for ameloblastomas (n=15; 93.8%), dentigerous cysts (n=42; 53.2%), and radicular cysts (n=134; 52.1%). For odontogenic keratocysts, swelling (n=19;38%) was also the first clinical presentation of the lesion, but the same proportion (38%) of lesions were detected incidentally during screening or routine radiographic examination. This was also the case for odontomas, where 55.6% of these lesions were detected incidentally on routine radiographs (n=5) (*P*<0.001).

Most of the lesions presented a radiolucent image, apart from odontomas which had a radiopaque appearance (*P*<0.001). Some lesions presented multilocular appearance, which was observed in 11 odontogenic keratocysts (22%), 4 ameloblastomas (26.7%) and one dentigerous cyst (1.3%). All radicular cysts presented a unilocular appearance (*P*<0.001). We found no differences between radiographic appearance of the solid ameloblastoma type (multilocular in 3 cases; 37.5%) and unicystic ameloblastoma (multilocular in one case; 14.3%) (*P*=0.310). Histological pattern of ameloblastomas were not related with radiographic appearance, with multilocular cases found in 3 (30%) plexiform and one (33.3%) mixed type (*P*=0.837). The involvement of an adjacent included tooth was seen in every case of dentigerous cyst and not observed in any cases of radicular cysts. It was detected in 14 odontogenic keratocysts (28%), in 5 (31.3%) ameloblastomas (4 of them in unicystic ameloblastomas) and 4 odontomas (44.4%) (*P*<0.001). Ameloblastomas and odontogenic keratocysts presented as larger sized lesion (5.2±1.7cm and 4.5 ±2.4cm, respectively) than dentigerous cysts (3.6 ±1.8cm), radicular cysts (2.8 ±1.3cm) or odontomas (2.8 ±1.2cm) (*P*<0.001). No differences on size were found between the solid or unicystic ameloblastomas (*P*=0.837) or between complex or compound odontomas (*P*=0.331) (Table 2).

Treatment

Surgical enucleation (with curettage) was the treatment of choice for most of the lesions in the sample. All cases of radicular cysts, dentigerous cysts, and odontogenic keratocysts were treated with this procedure. For ameloblastoma cases, partial resection surgery was carried out for two patients (12.5%) and total resection for another two patients. One case (11.1%) of a complex odontoma also underwent a partial resection (*P*<0.001).

- Analysis of recurrence-free time interval

We analysed the interval of time without recurrence for all patients during the follow-up period (mean follow-up time of 36.4±2.4 months ranging from 1 to 232 months). The overall mean recurrence-free interval was 185.1±8.3 months (95% CI of 168.9 to 201.3) with 88.2% of patients without recurrence at the 5-year follow-up mark.

We performed a univariate analysis using Kaplan-Meier curves method. The variables with significant association with higher number of recurrence cases were: anatomical site of the lesion (in mandible) (*P*=0.045), presence of multiple lesions at diagnosis (*P*=0.019), histological diagnosis of the lesions (especially for ameloblastomas and odontogenic keratocysts) (*P*=0.005), the presence of Gorlin-Goltz syndrome (*P*=0.006), the presence of multilocular radiographic appearance (*P*<0.001), and a radiographic size greater than 3.3cm (*P*=0.004) (Table 3).

To analyse the independent effect of each of these variables with significant results in univariate analysis we included them into a Cox regression analysis (Table 4). The diagnosis of the lesions was the only independent variable with a significant and independent prognostic value on the risk of recurrence (*P*=0.043), with ameloblastomas and odontogenic keratocysts presenting a HR of 6.1 (95% CI of 1.5 to 25.4, *P*=0.013) and 5.1 (95% CI of 1.3 to 20.4, *P*=0.023), respectively (Table 4).

However, when we adjusted the analysis with the treatment variable, the diagnosis of the lesion and the radiographic (radiolucent) appearance of the lesion presented a significant and independent value (*P*=0.01 and *P*=0.025, respectively).

**Table 2:**
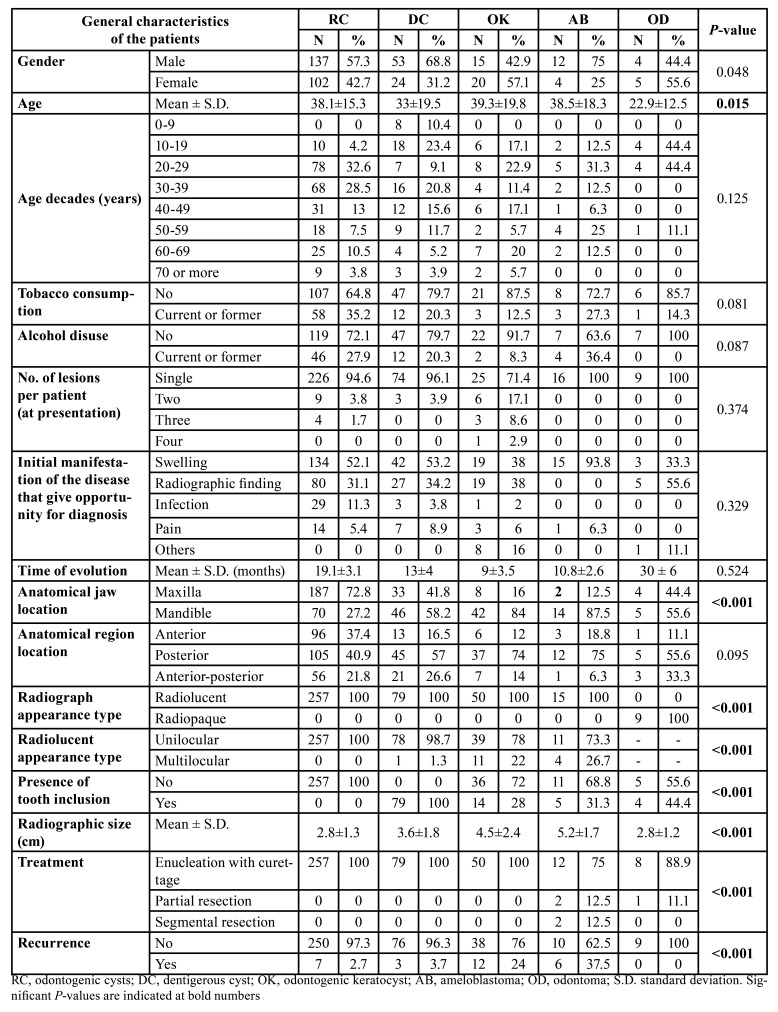
General characteristics of the five most common odontogenic cysts and odontogenic tumours.

**Table 3:**
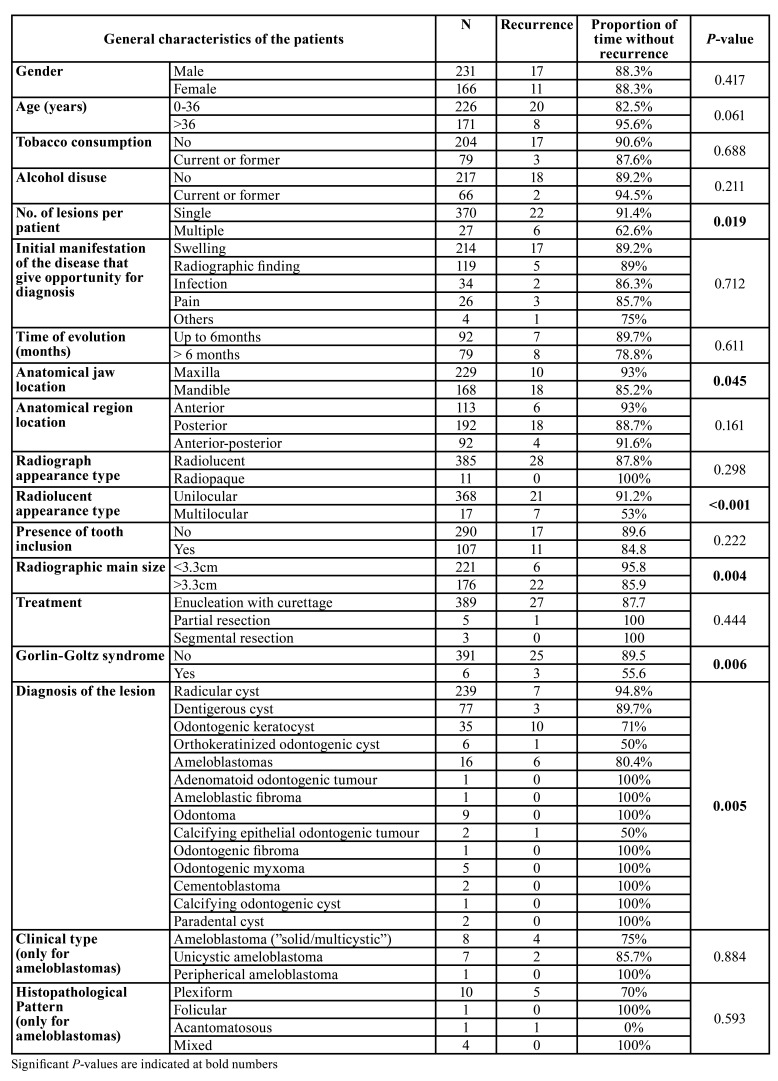
Univariable analysis of recurrence-free time interval of variables evaluated in the study.

**Table 4:**
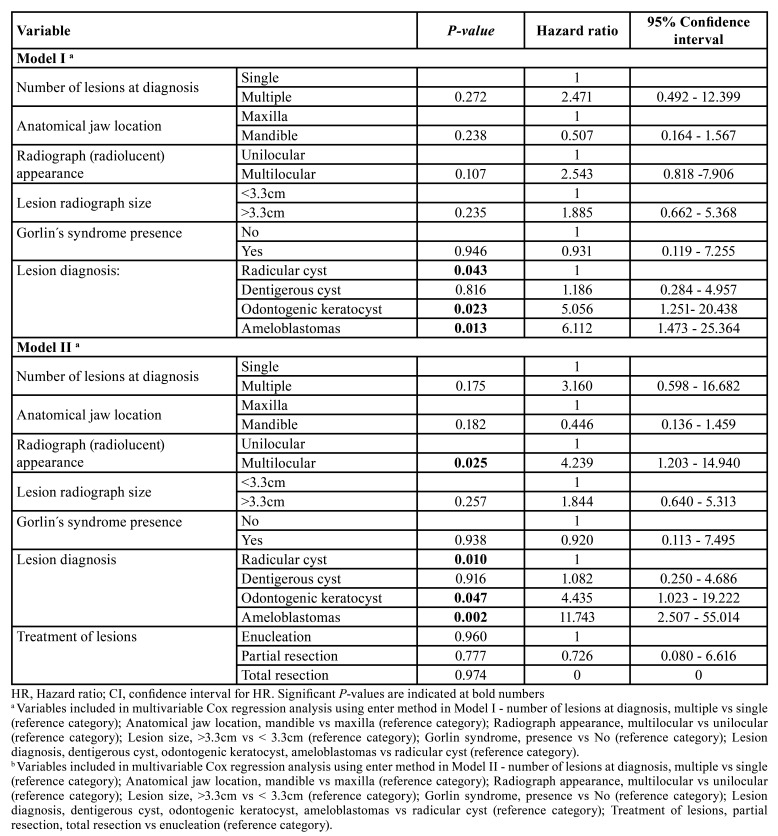
Multivariable analysis of recurrence-free time interval of variables with significant effect in univariable analysis.

## Discussion

We developed an observational study to analyse the clinical and pathological characteristics of a cohort of odontogenic cysts and tumours of the jaws in a Portuguese population, and using the new 2017 WHO classification system. We observed a predominant frequency of odontogenic cysts (91.5%) over odontogenic tumours. This is in line with studies and case series reporting on odontogenic cysts and tumours (7-9,15). In a Canadian population sample, Daley *et al*. (9) observed a higher relative incidence of odontogenic cysts (93.9%) over odontogenic tumours. In the Baghaei *et al*. (15) study, looking at an Iranian population cohort, this value corresponded to 86.4%.

Interestingly, if we use the WHO 2005 classification, where odontogenic keratocysts were considered neoplasms (KOT) and include them in the OT category, the proportion of odontogenic cysts in our sample drops to 79.7%. This leads to a proportional increase of odontogenic tumour frequency with KOT being the most frequent type of OT (56.8%). This is in line with other studies that showed an increased prevalence of OT when using the 2005 WHO classification (7,8), with KOT being the most common OT (6,8). With the new 2017 WHO classification, odontogenic keratocysts are considered odontogenic cysts contributing to a lower prevalence of OT as observed in several studies (9,16,17), except for some studies with African cohorts.

The third decade of life was the most affected decade, for both odontogenic cysts and tumours, which correlates with observations in other studies also (5,18-20). We observed a higher number of cases in males, without a significant difference between OC and OT. This gender distribution was also observed in other studies on odontogenic cysts (1,3,15,18,19,21-25) and odontogenic tumours (5,20,26). Odontogenic cysts occurred more often in the maxilla compared to odontogenic tumours that occurred more commonly in the mandible. This is in line with findings of other population cohorts on odontogenic cysts (3,15,17,19,24,27) and on odontogenic tumours (5,8,14,20,26,28).

The lesion most commonly observed in our sample was the radicular cyst. This was followed by dentigerous cysts, odontogenic keratocysts, ameloblastomas and odontomas. These lesions represented 95% of the sample. This frequency distribution is similar to that reported in other studies analysing OC and/or OT (3,16-19,21-23,25,27,29).

Radicular cysts, the most frequent lesion in our sample, corresponded to 58% of all cases, affecting slightly more males and occurring most commonly in the third decade of life. This result corroborates with those of other publications in frequency, age and gender (3,18,19,21). Nevertheless, there is a difference in the frequency rate of radicular cysts between different countries, ranging from 40% to 80%. Some authors (3,24) have associated the higher frequency of these lesions to poor oral health conditions and lower socio-economic classes, which shows the importance of promoting better oral health worldwide. Residual cysts were included in the radicular cyst diagnosis according to the 2017 WHO classification. However, if analysed alone, they correspond to the 4th most common lesion in our sample as observed in other studies (3,17,21-23,25,27,29).

Dentigerous cysts corresponded to the second most commonly occurring lesion in our sample as also noted by other studies. However, the study by Baghaei *et al*. (15) evaluated 70 odontogenic cysts and found that dentigerous cysts were the most commonly occurring lesion. Most studies confirm the young age at which this type of cysts presents. This is in line with our results with dentigerous cysts mostly occurring in patients in the second decade of life, and being the only lesion with cases in the first decade of life (13,17,22,24). More cases were present in the posterior region of the mandible, similar to the observations made by other studies (1,12,24,25,29). Using the 2017 WHO classification, eruption cysts, which were found in 5 cases in our sample (4 of them in the first decade of life), were included in the dentigerous cyst diagnosis, due to their aetiological nature.

Odontogenic keratocysts were the third most common lesion in this group. It has been suggested that this lesion may occur more frequently in the western world and amongst Caucasian patients, as opposed to in Asian and African regions or amongst patients of black African descent (20). A higher relative frequency of odontogenic keratocysts (or KOT, using the 2005 WHO classification) compared with ameloblastomas was reported in Mexico (7) and Brazil (6,8), compared with a higher frequency of ameloblastomas in Egypt (28), Nigeria (20,26), and South Africa (30). This can however be biased, as suggested by Oginni *et al*. (20), as in some developing countries patients may seek help or present to healthcare centres only in severe cases, for instance in the case of a severe swelling. Interestingly, we observed that odontogenic keratocysts were the most common type of odontogenic cyst being detected by screening radiographs (38%), as opposed to ameloblastomas that were detected mainly due to the clinical manifestation of swelling (94%). We found a slightly higher number of cases in females, as also reported by other authors (1,2,13) but not all (12,20,23) and with a peak in the third decade of life, similar to the results reported by Selvamani *et al*. (25). Odontogenic keratocysts predominantly affected the mandible and the posterior region, confirming the well documented predilection of the mandible (1,12,13,17,20,23,25-27,29).

Odontogenic keratocysts were the lesions that presented with more synchronic or multiple cysts at the time of the diagnosis. Moreover, we observed an association with Gorlin-Goltz syndrome in 6 patients (17%). They all presented with multiple odontogenic keratocysts and other manifestations of the syndrome such as bone/skeletal alterations, basal cell nevus and/or carcinomas or familial history of the disease as described by others. Unfortunately, very few studies with large cohort of OC/OT have evaluated this syndrome. Del Corso *et al*. (12), evaluating an Italian cohort of 1136 jaw cysts, found only 2 cases of this syndrome amongst the 16 odontogenic keratocysts in the sample (12.5%). In a Turkish sample of 452 odontogenic cysts, Açikgöz *et al*. (1), found one case of this syndrome amongst the 15 odontogenic keratocysts (7%), and Kambalimath *et al*. (18) found no cases in their study of 150 odontogenic cysts in an Indian population. Our higher frequency in comparision with these studies, could be related with the familial cases that we found, or perhaps by the exhaustive and multidisciplinary clinical examination performed in the patients of this central hospital. In view of this, we suggest an integrative and multidisciplinary diagnostic plan for all suspected or confirmed cases of odontogenic keratocysts, including also a comprehensive personal and family clinical history. Although most of the odontogenic keratocysts in this sample had a unilocular radiographic appearance, the multilocular pattern was present in 22% of these cysts, representing 2.7% of all odontogenic cysts, which is in line to the proportion reported by other studies (1) suggesting an aggressive pattern for some of these odontogenic keratocysts.

As the most frequently occurring odontogenic tumour in our cohort of patients, ameloblastoma cases were more common in male patients and in the third decade of life, similar to the observations by others authors (5,14,20,26). There was a clear predominant occurrence in the mandible (88%) and particularly in the posterior region (75%), with similar values to that of odontogenic keratocysts (84 and 74%, respectively). This predilection to the mandible was also reported by other authors looking at odontogenic tumour lesions (10,14,16,26,28,30). Ameloblastomas were the lesions with more cases of multilocular radiographic appearance (26%), which is in accordance with the literature and with values close to those also observed in odontogenic keratocysts, showing the aggressive character of these lesions.

Analysing the histological characteristics of these lesions, we observed that more than half of ameloblastomas presented a plexiform pattern, as observed by Fernandes *et al*. (16) in 55% of cases. However, Oginni *et al*. (20) found more cases of follicular pattern (41%). The most common macroscopic type of ameloblastoma corresponded to the “solid type”, as also observed by Fernandes *et al*. (16). Nevertheless, our values for unicystic ameloblastomas are higher than that reported by this last study (17%).

Odontomas were the 2nd most common odontogenic tumour, similar to the description by Fernandes *et al*. (16). However, the prevalence of odontomas has shown great variations over the world with high frequency in American and European countries (6,9,11,17) and low in Africa and Asian countries (5,14,20,26,30); interestingly this is the opposite of the pattern observed for ameloblastomas (5). These differences, more than geographic variations, could be related with the hamartomatous nature of these lesions, with few clinical manifestations that could lead to a lower rate of treatment of these lesions and a lower rate of histopathological analysis (5). In fact, most of the odontomas in our sample were detected incidentally on radiographs and not by clinical symptoms. This could suggest that the frequency of odontomas could be underestimated in many reports.

Unfortunately, data on the recurrence of odontogenic cysts (2,12,13) and odontogenic tumours has been very scarce (10,11,14). Furthermore, we did not find any study using longitudinal analysis of the recurrence-free time interval. We note that the recurrence was significantly related with some lesions such as ameloblastomas (37.5%) and odontogenic keratocysts (24%), and less with dentigerous cysts (3.8%), and radicular cysts (2.7%), which is in line with the existing literature reporting recurrence (10,12). However, we also aimed to evaluate the influence of clinical and pathological variables on the recurrence, performing a multivariate analysis on a longitudinal approach. In multivariate analysis, the diagnosis of the lesions was the only independent variable, with ameloblastomas and odontogenic keratocysts presenting a hazard ratio of 6.1 and 5.1, respectively. In the univariate analysis, we did not obtain a positive association with the treatment, although we acknowledge that it could be biased according to the diagnosis of the lesion, as for example, bone resections were more common in odontogenic tumours. Therefore, we included the treatment variable in a second model of multivariable analysis and observed that the diagnosis and the radiographic appearance of the lesions presented a significant and independent value. This confirms not only the importance of the histological diagnosis, but also the importance of a longer follow-up for these lesions, in particular for ameloblastomas and odontogenic keratocysts.

Despite the fact that odontogenic keratocysts were included again in the group of odontogenic cysts, they do have some aggressive characteristics which are similar to characteristics associated with some odontogenic tumours (such as ameloblastomas). These characteristics involve recurrence rate, multilocular radiographic appearance, anatomical location, or large dimension of the lesion. Interestingly, we did not find a significant difference with the histological pattern of the ameloblastomas and also for the macroscopic type of ameloblastomas. This suggests that, although unicystic ameloblastoma presents clinically as a cystic lesion, it should be regarded as a true odontogenic tumour and a member of the ameloblastoma group.

We acknowledge some limitations in our work, many of them related with the retrospective nature of this study, and others related with rare presentation of some lesions that do not allow us to make statistical comparisons with more groups of lesions. Also, the treatment, as expected, was probably related to the severity of the lesions which could cause some bias in the treatment analysis. Moreover, some more recent treatment approaches were not analysed as these were not usually performed during the analysed period of time. We intend in future to evaluate the treatment options in a selected group of homogeneous lesions and also to analyse the value of some molecular biomarkers as adjuvant tools in the diagnosis and prognosis of these lesions.

In conclusion, radicular cysts were the most commonly occurring type of odontogenic cyst and ameloblastomas were the most commonly occurring type of odontogenic tumour in this Portuguese population cohort. The highest recurrence rate was noted amongst ameloblastomas and odontogenic keratocysts. This large sample provides useful information about the frequency distribution of odontogenic cysts and odontogenic tumours over a period of 18 years allowing valuable comparison with other countries.
